# Supplementary figures and images for: Exploratory Metabolomic and Lipidomic Profiling in a Manganese-Exposed Parkinsonism-Affected Population in Northern Italy
Source: Metabolites. 2025 Jul 20;15(7):487. doi: 10.3390/metabo15070487 (PMC12299838; doi:10.3390/metabo15070487)

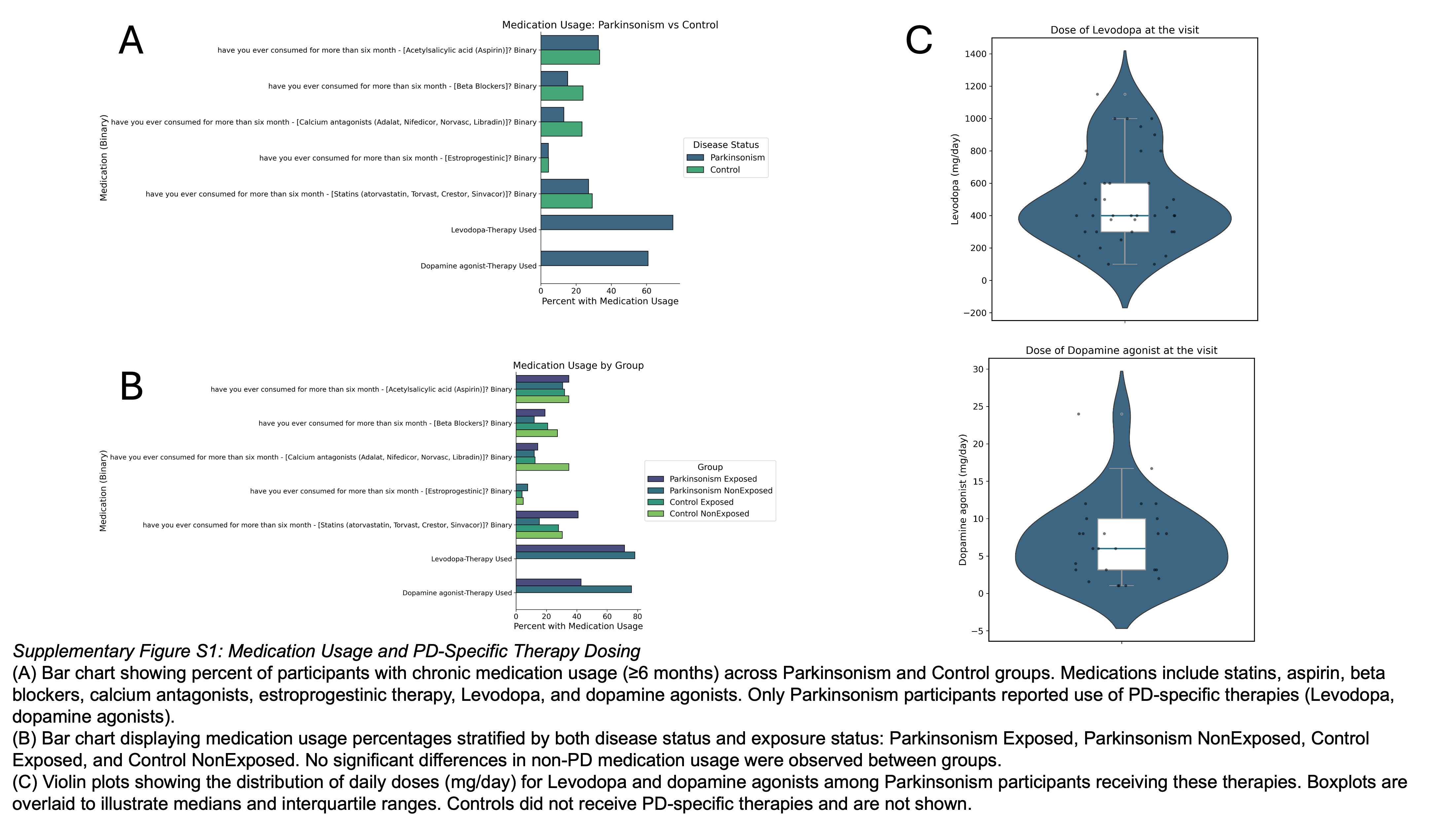

Supplement: Supplementary file 1 [file metabolites-15-00487-s001.zip › Supplemental_Figure_S1_SF1.png]

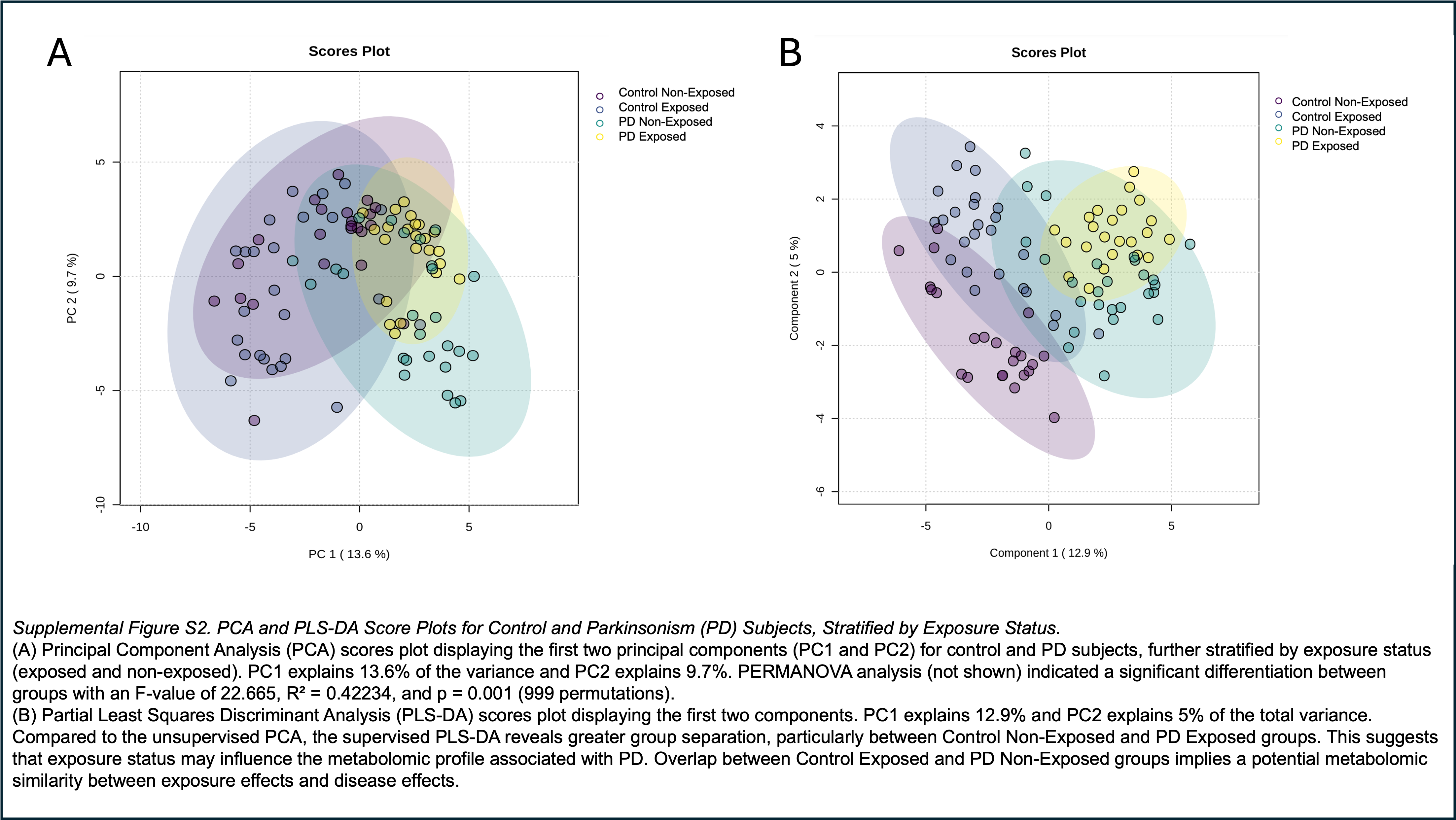

Supplement: Supplementary file 1 [file metabolites-15-00487-s001.zip › Supplemental_Figure_S2_SF2.png]

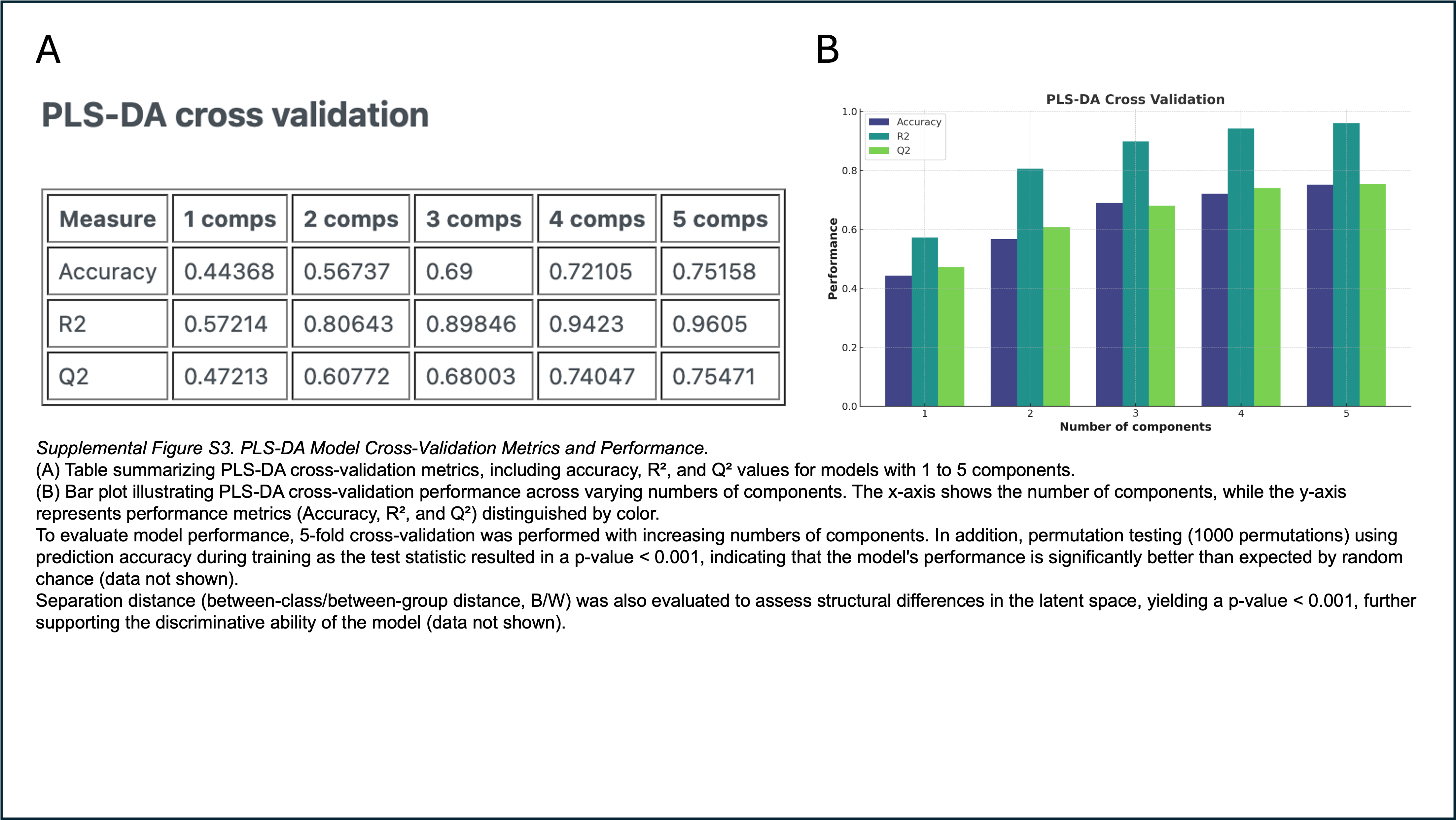

Supplement: Supplementary file 1 [file metabolites-15-00487-s001.zip › Supplemental_Figure_S3_SF3.png]

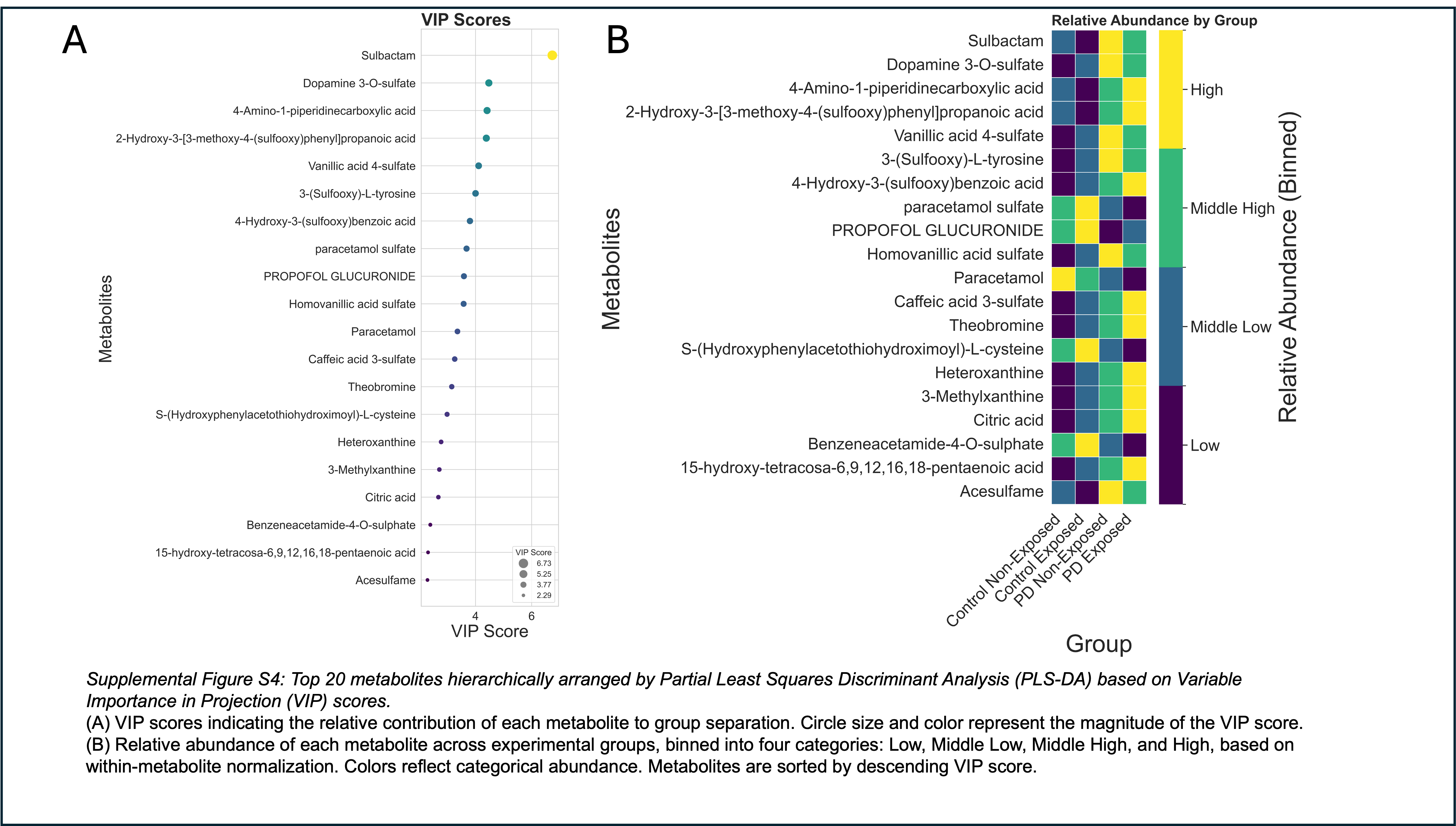

Supplement: Supplementary file 1 [file metabolites-15-00487-s001.zip › Supplemental_Figure_S4_SF4.png]

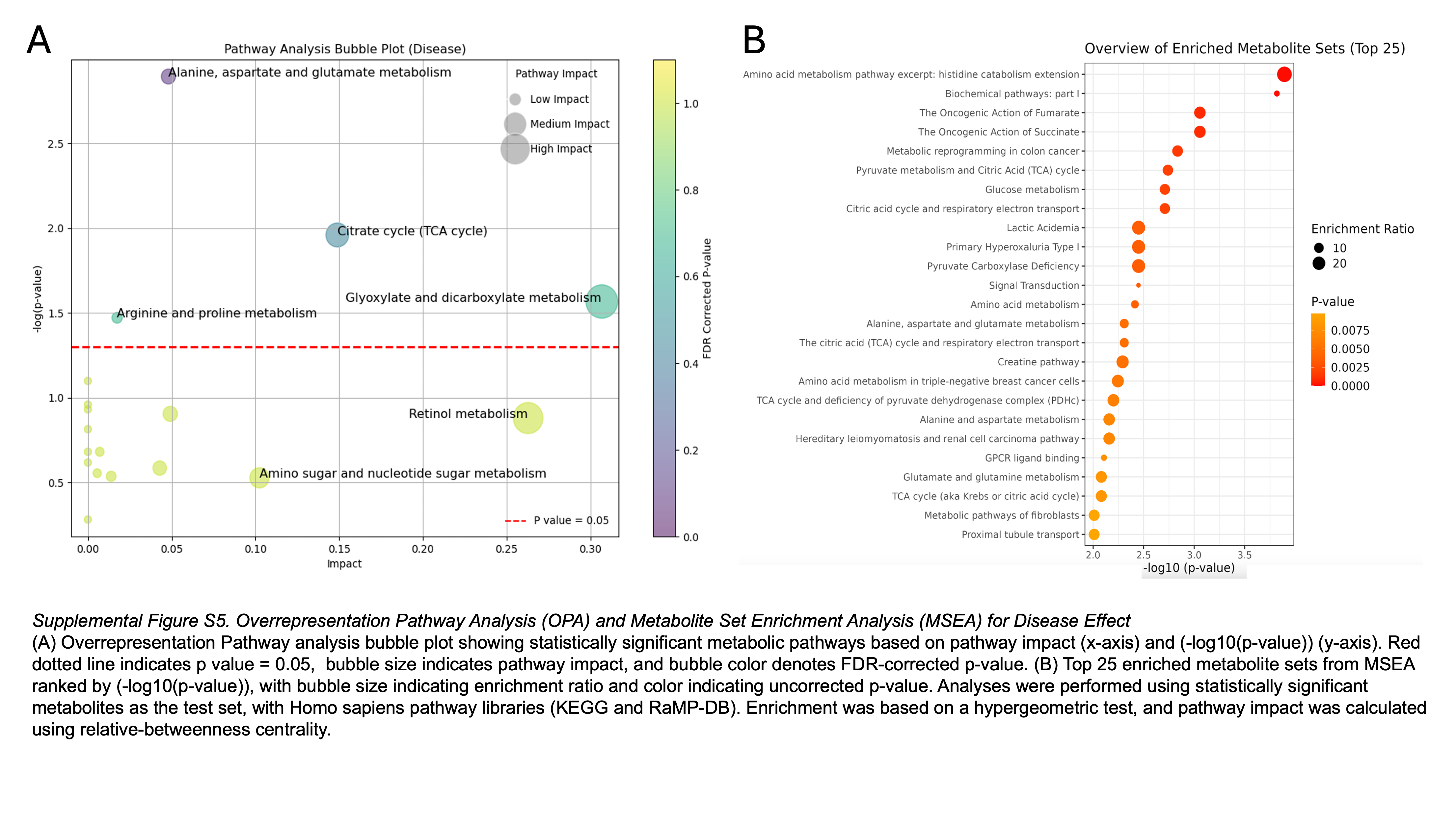

Supplement: Supplementary file 1 [file metabolites-15-00487-s001.zip › Supplemental_Figure_S5_SF5.png]

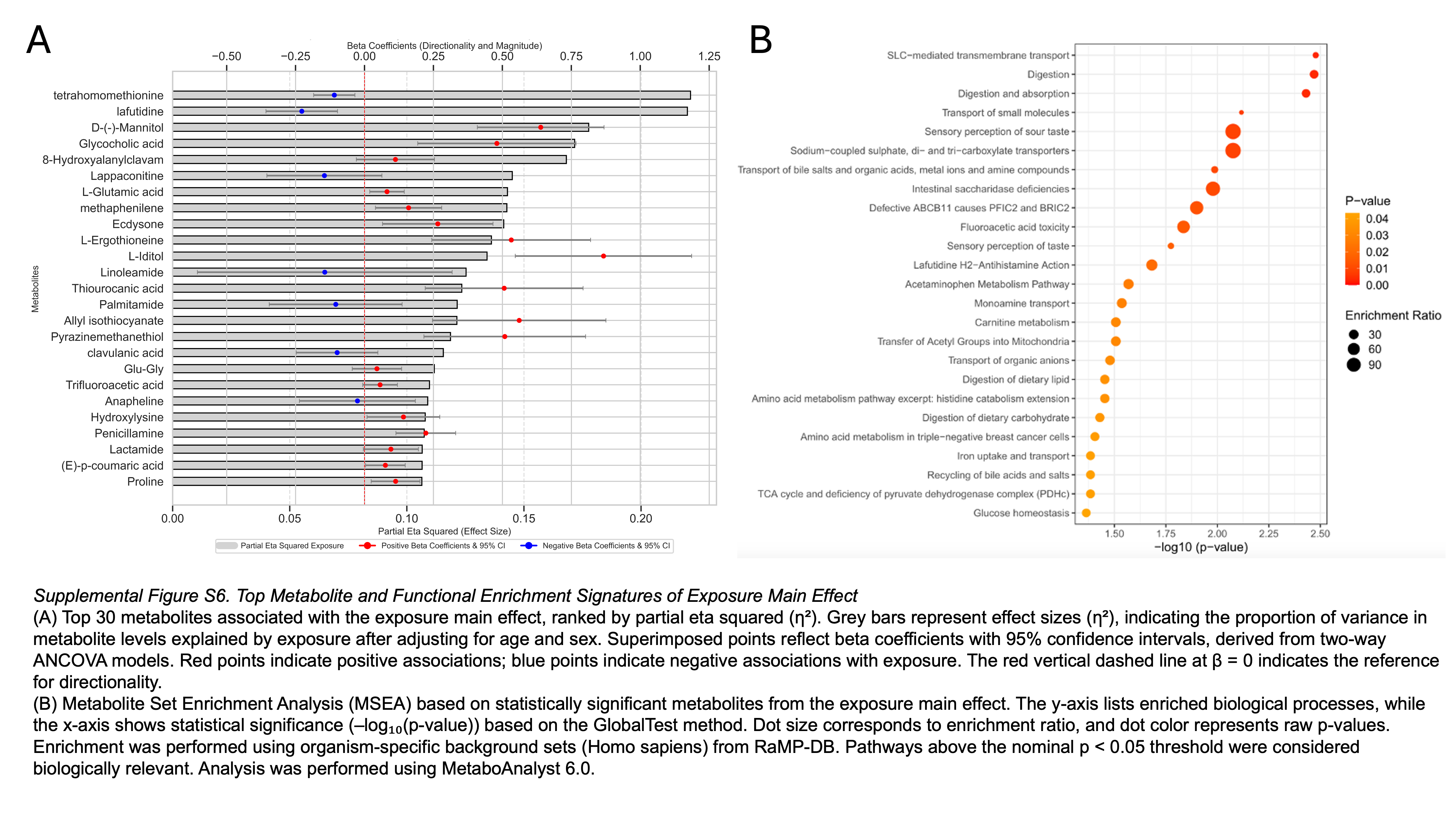

Supplement: Supplementary file 1 [file metabolites-15-00487-s001.zip › Supplemental_Figure_S6_SF6.png]

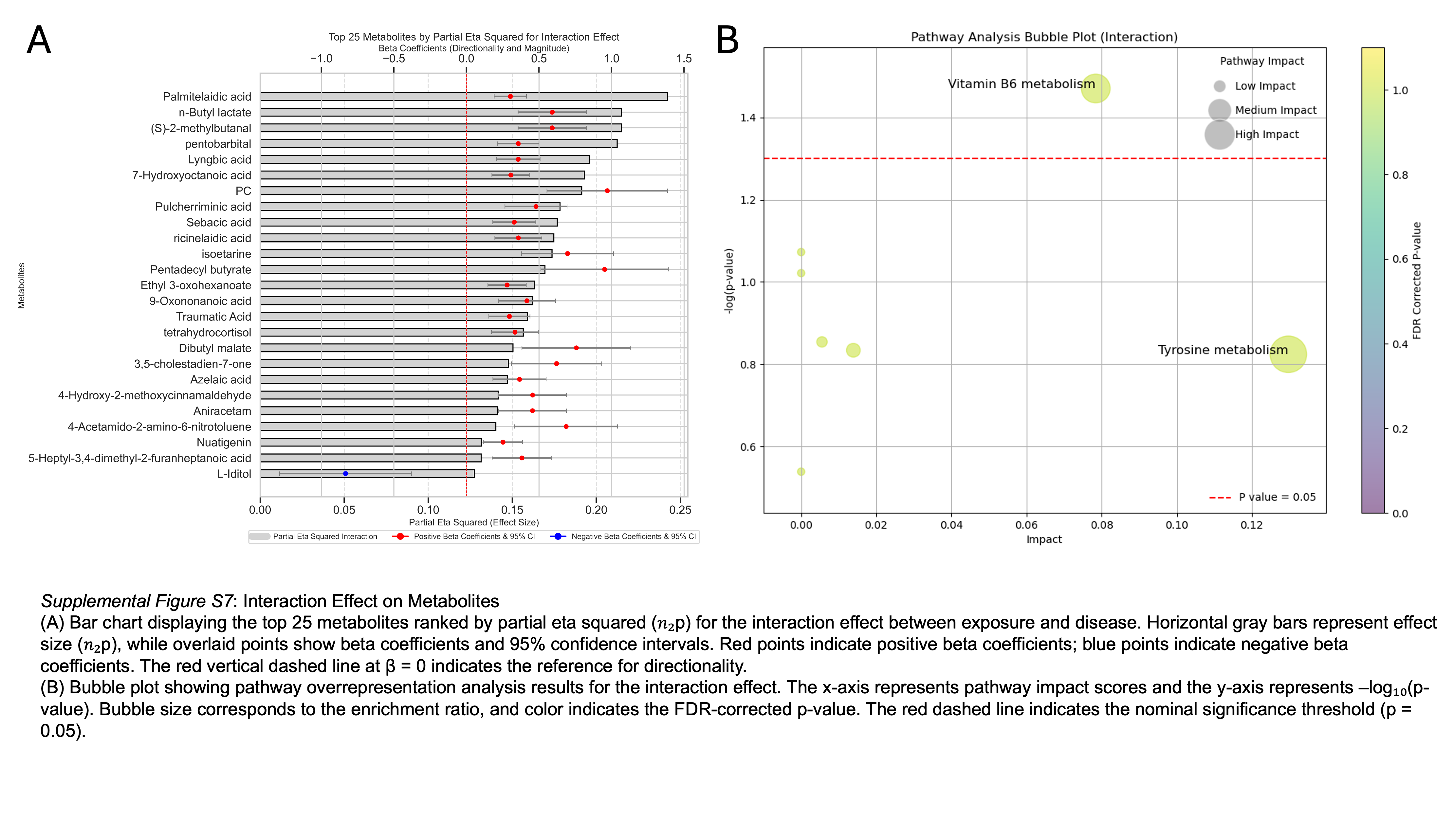

Supplement: Supplementary file 1 [file metabolites-15-00487-s001.zip › Supplemental_Figure_S7_SF7.png]
